# Calibration of Planar Reflectors Reshaping LiDAR’s Field of View

**DOI:** 10.3390/s21196501

**Published:** 2021-09-29

**Authors:** Michał Pełka, Janusz Będkowski

**Affiliations:** 1 Tooploox, 53-601 Wrocław, Poland; michal.pelka@tooploox.com; 2 Institute of Fundamental Technological Research, Polish Academy of Sciences, 02-106 Warsaw, Poland

**Keywords:** LiDAR, ICP, mapping, calibration, reshape field of view, solid state LiDAR

## Abstract

This paper describes the calibration method for calculating parameters (position and orientation) of planar reflectors reshaping LiDAR’s (light detection and ranging) field of view. The calibration method is based on the reflection equation used in the ICP (Iterative Closest Point) optimization. A novel calibration process as the multi-view data registration scheme is proposed; therefore, the poses of the measurement instrument and parameters of planar reflectors are calculated simultaneously. The final metric measurement is more accurate compared with parameters retrieved from the mechanical design. Therefore, it is evident that the calibration process is required for affordable solutions where the mechanical design can differ from the inaccurate assembly. It is shown that the accuracy is less than 20 cm for almost all measurements preserving long-range capabilities. The experiment is performed based on Livox Mid-40 LiDAR augmented with six planar reflectors. The ground-truth data were collected using Z + F IMAGER 5010 3D Terrestrial Laser Scanner. The calibration method is independent of mechanical design and does not require any fiducial markers on the mirrors. This work fulfils the gap between rotating and Solid-State LiDARs since the field of view can be reshaped by planar reflectors, and the proposed method can preserve the metric accuracy. Thus, such discussion concludes the findings. We prepared an open-source project and provided all the necessary data for reproducing the experiments. That includes: Complete open-source code, the mechanical design of reflector assembly and the dataset which was used in this paper.

## 1. Introduction and Related Work

LiDAR (light detection and ranging) in general is the method for determining ranges (variable distance) between an object and a laser by measuring the time it takes for reflected light to return to the receiver. This measurement instrument is mainly used for the 3D digitization of the urban environment, cultural heritage and archaeology, underground environment, environmental monitoring, forestry and agriculture [1]. The purpose of the study was to verify an experimentally proposed novel calibration process capable of preserving LiDAR’s accuracy and range after reshaping the FOV (Field Of View) with planar reflectors. Base on our best knowledge such work has not yet been elaborated in the literature. We use Livox Mid-40 (manufacturer Livox, Hong Kong, China, https://www.livoxtech.com/): The robotic LiDAR sensor based on incommensurable scanning that allows straightforward mass production and adoption in autonomous robots [2]. There are many types of LiDAR applications such as Terrestrial Laser Scanning, which uses a highly accurate measurement instrument that works in the so-called stop-scan fashion looking from a mobile robotics point of view [3]. This means that the robot stops at certain goals to acquire highly accurate 3D measurements. Another family of applications is mobile mapping systems composed of highly accurate planar LiDARs, or 3D multi-beam lasers such as Velodyne. An overview of scanning and reconstruction methods is discussed in Lehtola et al. [4]. Recently, the development of Solid-State LiDARs shows the potential reduction of such devices. Typical data produced by LiDAR are measurements of distance and reflectance; thus white reflective objects such as lines or signs can be identified easily. An important aspect of the multi beam LiDAR is the intrinsic calibration [5,6,7]. Such calibration is crucial in mobile mapping applications where the measurement instrument is heavily occupied and works assuming diverse environmental conditions. The gap between rotating and Solid-State LiDARs is evident since the field of view of Solid-State LiDARs is still limited. Review [8] shows an extensive overview of MEMS (Micro Electro Mechanical Systems) scanning mirrors specifically for applications in LiDAR systems. Such technology will improve Solid-State LiDARs but it requires a sophisticated laboratory and the time from the design to the delivery is rather a barrier in fast prototyping. For this reason, the investigation of reshaping the field of view of the LiDAR with planar reflectors is the main topic of the paper. Many other researchers proposed different methods incorporating the rotation of the LiDAR [9,10]. In such cases, the rotation of the LiDAR requires additional mechanics and electronics. For such approaches the synchronization of the LiDAR data with GPS (Global Positioning System) and odometry is crucial. This leads our research to reduce the number of additional mechanical and electronic parts for reshaping LiDAR’s field of view. Therefore, the overall design is simplified and the final prototype is more affordable compared with competitive solutions.

Reshaping the field of view of the sensors is an interesting research topic since it allows to customize a specific application for certain needs. For example, authors Endres et al. [11] introduced the combination of an RGB-D camera with two planar mirrors to split the field of view. It covers both the front and rear views of a mobile robot. They describe how to estimate the extrinsic calibration parameters of the modified sensor using a standard parametrization. For solving the graph SLAM (simultaneous localization and mapping) optimization problem the g2o framework [12] is used. More information concerning the graph SLAM can be found in [13,14,15,16]. An alternative approach is discussed in Akay et al. [17] where a proposed solution employs mirrors to introduce virtual RGB-D cameras into the system. The proposed system does not have any space limitations, data bandwidth constraints or synchronization problems and it is more affordable since it does not require extra cameras. The authors developed formulations for the simultaneous calibration of real and virtual RGB and RGB-D cameras and provided methods for 3D reconstruction from these cameras. It is worth mentioning that RGB-D mapping and 3D reconstruction have been intensively researched topics in recent years. An interesting comparative study of registration methods for RGB-D video of static scenes is discussed in Morell-Gimenez et al. [18].

The authors Aalerud et al. [19] proposed a method for the simulation and design of a radially reshaped field of view. This work shows great potential for an increased number of usable measurements. Unfortunately, the authors did not address the problem of decreased accuracy related to the not ideal geometrical placement of the reflectors. Moreover, it is difficult to find relevant discussion in the literature [20]; therefore, we address this problem by providing an end-to-end framework for the calibration of the geometric placement of the reflectors. Adding reflectors can improve the mobile robotic perception [21] by augmenting planar 2D measurements; thus, Unmanned Aerial Vehicles can easily navigate in indoor environments. Mirrors can be used for extending the LiDAR field of view in self-driving vehicles [22]. Generally, it is easy to imagine plenty of applications requiring changing the LiDAR field of view, e.g., security applications, autonomous driving, etc. For this reason, we propose a general framework that can be used for other researchers in developing a specific LiDAR field of view configuration.

This topic is relevant looking at recent developments in mobile robotics such as SLAM and search and rescue applications where reconstructing 3D scenes is one of the goals of the autonomous machine and autonomous cars that will use a predefined map for localization purposes [23,24]. Obviously, it is evident that autonomous cars can collect data and contribute to global map updates; thus, we cope with a large-scale problem that has recently been addressed by many researchers. The term SLAM [25,26] corresponds to the so-called “chicken and egg dilemma”—what was first: The chicken or the egg? Therefore we should have a proper map representation that is compatible with observations derived from sensors to localize within this map, and we need accurate localization to build the map. In the literature [12], the SLAM problem is divided into so-called front-end and back-end. The front-end is typically responsible for initial trajectory generation and the back-end is responsible for so-called loop closing. Loop is the situation when the measurement instrument visits the same location as visited some time ago, assuming a continuous trajectory between these two time intervals. The back-end is typically solved using a so-called graph-based technique that minimizes the error between the observed and desired relative pose between trajectory/trajectory nodes. Due to the large scale aspect of SLAM, it can be mentioned that recent research shows an interest in cloud-based calculations of back-end [25,27,28].

The objective and at the same time the rationale addressing the research problem is lowering the cost of LiDAR sensors. This is related to reshaping the field of view, which can be done with planar reflectors. Such an approach requires additional calibration due to the fact of assembly and manufacturing imperfections. Moreover, some applications such as autonomous mobile robots equipped with such sensors will deal with the problem of self-calibration during daily operations. For this reason, we propose a novel calibration process and an open-source approach. It fulfils the gap in affordable LiDAR sensors with a reshaped field of view. Based on our best knowledge such an approach has not yet been elaborated in the literature; many studies even used additional planar reflectors for LiDARs. Our future work will be investigating the usage of presented LiDAR sensors for mobile robot localization; therefore, this research contributes to affordable mobile robotics.

## 2. Materials and Methods

### 2.1. Mechanical Design

**Figure 1:**
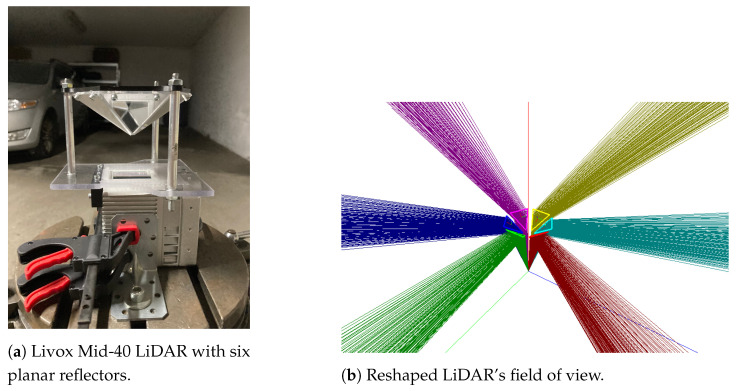
Prototype of LiDAR with planar reflectors.

**Figure 2:**
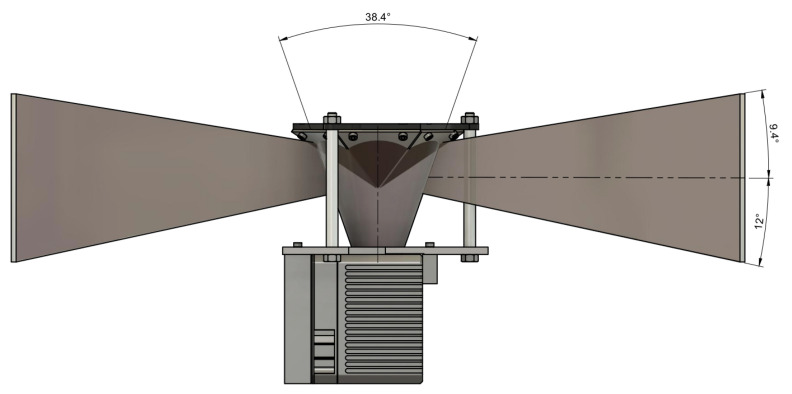
Reshaped field of view; plot of vertical situation.

**Figure 3:**
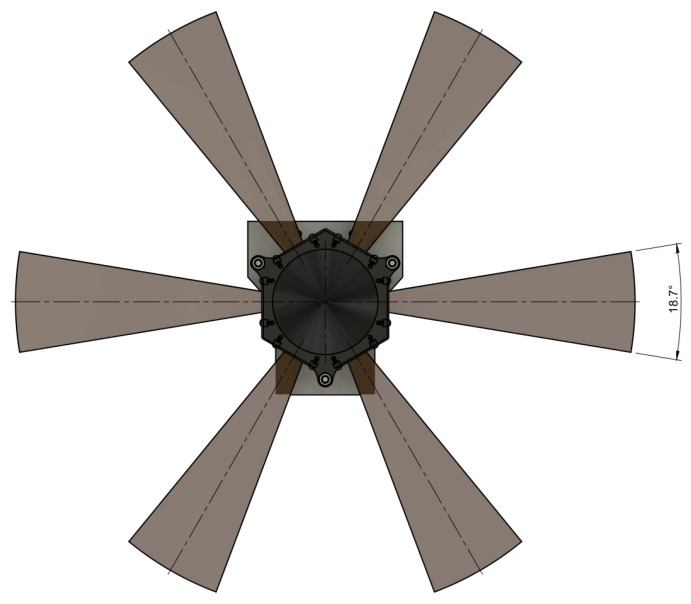
Reshaped field of view; plot of horizontal situation.

The Livox Mid-40 LiDAR has an originally conical field of view with the apex in the optical centre and an apex angle equal to 38.4 degrees. Relevant parameters of this LiDAR [29] are shown in Table 1. This table shows also Z + F IMAGER 5010 [30] parameters as a reference ground-truth source. Figure 1 shows a prototype used in experiments and simulations of the reshaped field of view. The field of view after modification consists of six segments that have properties:

- The field of view (vertical) spreads from −12${}^{{}^{\circ}}$ to 9.4${}^{{}^{\circ}}$ (Figure 2).
- The field of view (horizontal) has six segments orientated radially with an angle up to 18.7${}^{{}^{\circ}}$ (Figure 3).

**Figure 4:**
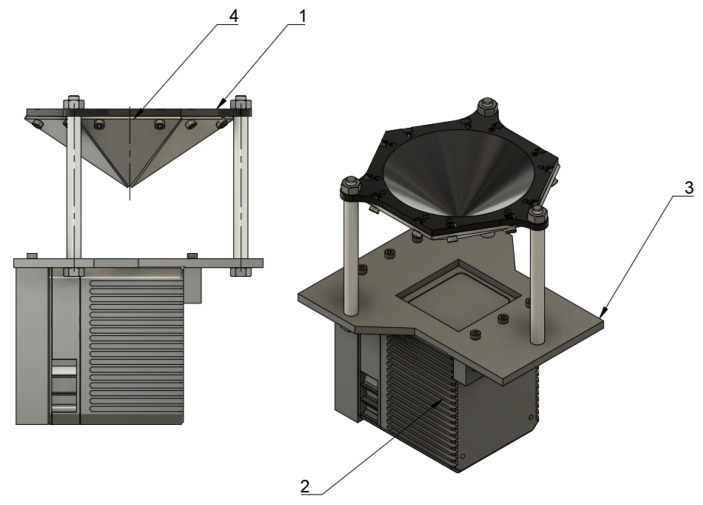
Three-dimensional CAD model of assembly. 1—hexagonal pyramid; 2—Livox LiDAR; 3—face plate; 4—one of six mirrors.

Due to the limited precision of the mirror assembly (the main contributor is the thickness of the top PMMA (PolyMethyl MethAcrylate) surface of the mirror, the usable angular range is limited. Therefore, measurement outliers are evident when a given ray is assigned to the wrong mirror. The reflector assembly was designed using the Autodesk Fusion 360 software [31]. It is CAD (Computer Aided Design) software that helps in the mechanical design process. It allows producing 3D models for 3D printing and necessary documentation for further manufacturing. The designed structure consists of the main chassis (which resembles a hexagonal pyramid) with a set of mirrors screwed into the pyramid’s faces. The screws’ holes are placed outside the useful surface of the mirror (marked as 1 in Figure 4). The main chassis was 3D printed using a consumer-grade FDM (Fused Deposition Modeling) printer with PLA (PolyLactic Acid) filament. The mirrors’ shape is obtained with a CNC plotter from the PMMA sheet with the reflective bottom surface (marked as 4 in Figure 4). The original laser scanner has a front plate attached. The front plate (marked as 3 in Figure 4) has a rectangular hole for lasers’ scanner optics and multiple holes for assembly—ϕ 3 mm for mounting laser scanner and ϕ 6 mm for three pillars. The hexagonal pyramid is attached to pillars.

### 2.2. Calibration Method

Due to the imperfect assembly of the planar reflectors, the metric measurement is poor. To solve this issue the calibration method is designed and implemented as an open-source project [32]. It is composed of observation equations implemented with Ceres solver [33]. It is based on the commonly used Iterative Closest Point procedure [34,35,36]. The calibration method is independent of mechanical design and does not require any fiducial markers on the mirrors like in Chen et al. [22].

#### 2.2.1. Data Acquisition

**Figure 5:**
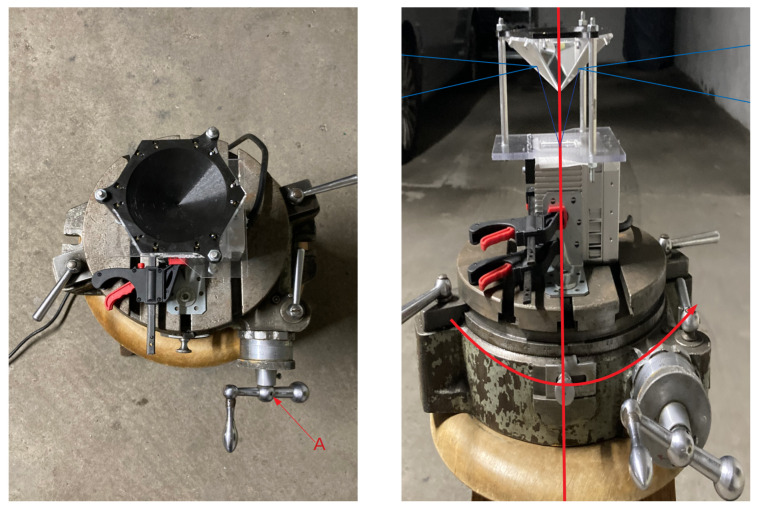
Experimental prototype mounted onto precise rotating table during data acquisition procedure. A—axis for changing rotation angle.

Due to six planar reflectors, it is advised to use stop-scan fashion in different locations assuming additional rotation of the measurement instrument around its axis (Figure 5). For this purpose we used a precise rotating table; therefore, we collected 36 static measurements for each measurement station by rotating the table by 10 degrees. Each static measurement consists of three seconds of the recorded Livox Mid-40 data. In such a scheme of data acquisition, there is a sufficient number of overlapping data of different mirrors. Additionally, the entire scene was scanned using Terrestrial Laser Scanning measurement instrument Z + F IMAGER 5010 (manufacturer: Z + F, 88239 Wangen, Germany, https://www.zofre.de/) (range uncertainty of 1 mm [30]). This ground-truth data is an order of magnitude more accurate than the experimental prototype.

#### 2.2.2. Iterative Closest Point

Local point $P^{l}$($x^{l}$,$y^{l}$,$z^{l},1$) is represented in Euclidean space as the point in local reference frame. The matrix [**R**, *T*] is the transformation of local point $P^{l}$ into point $P^{g}$($x^{g}$,$y^{g}$,$z^{g}$) in global reference frame; thus

(1) $$P^{g}=\left[R,T\right]P^{l}$$

(2) $$\Psi_{\left[R^{j},T^{j}\right]}\left(R^{j},T^{j},x_{i}^{l,j},y_{i}^{l,j},z_{i}^{l,j}\right)=\left(x_{i}^{g,j},y_{i}^{g,j},z_{i}^{g,j}\right)=\left(\begin{array}{cccc} r_{11}^{j} & r_{12}^{j} & r_{13}^{j} & t_{1}^{j}\\ r_{21}^{j} & r_{22}^{j} & r_{23}^{j} & t_{2}^{j}\\ r_{31}^{j} & r_{32}^{j} & r_{33}^{j} & t_{3}^{j} \end{array}\right)\left(\begin{array}{c} x_{i}^{l,j}\\ y_{i}^{l,j}\\ z_{i}^{l,j}\\ 1 \end{array}\right)$$

(3) $$\begin{array}{c} \underset{residuals}{\underbrace{\left[\begin{array}{c} x^{\delta }\\ y^{\delta }\\ z^{\delta } \end{array}\right]}}=\underset{target\;values}{\underbrace{\left[\begin{array}{c} 0\\ 0\\ 0 \end{array}\right]}}-\underset{model\;function}{\underbrace{\left(\begin{array}{c} \Psi_{\underset{\beta^{j}}{\underbrace{\left[R^{j},T^{j}\right]}}}\left(R^{j},T^{j},x^{l,j},y^{l,j},z^{l,j}\right)-\Psi_{\underset{\beta^{k}}{\underbrace{\left[R^{k},T^{k}\right]}}}\left(R^{k},T^{k},x^{l,k},y^{l,k},z^{l,k}\right) \end{array}\right)}} \end{array}$$

Equation (2) transforms the $i^{th}$ local point $\left(x_{i}^{l,j},y_{i}^{l,j},z_{i}^{l,j},1\right)$ in $j^{th}$ [**R**, *T*] into global reference system $\left(x_{i}^{g,j},y_{i}^{g,j},z_{i}^{g,j}\right)$; therefore, it can be used for building point to point observation Equation (3), incorporating two poses $\left[R^{1},T^{1}\right]$ and $\left[R^{2},T^{2}\right]$, where ${\left[\begin{array}{ccc} x^{\delta } & y^{\delta } & z^{\delta } \end{array}\right]}^{⊺}$ are residuals, ${\left[\begin{array}{ccc} 0 & 0 & 0 \end{array}\right]}^{⊺}$ are target values and $\Psi_{\left[R^{j},T^{j}\right]}\left(R^{j},T^{j},x^{l,j},y^{l,j},z^{l,j}\right)-\Psi_{\left[R^{k},T^{k}\right]}\left(R^{k},T^{k},x^{l,k},y^{l,k},z^{l,k}\right)$ is the model function. The Iterative Closest Point optimization problem for point to point observations is defined as Equation (4), where there are C pairs of points contributing to the optimization process.

(4) $$\min_{R^{j},T^{j},R^{k},T^{k}}\sum_{i=1}^{C}{\left(\left[\begin{array}{c} 0\\ 0\\ 0 \end{array}\right]-\left(\begin{array}{c} \Psi_{\left[R^{j},T^{j}\right]}\left(R^{j},T^{j},x_{i}^{l,j},y_{i}^{l,j},z_{i}^{l,j}\right)-\Psi_{\left[R^{k},T^{k}\right]}\left(R^{k},T^{k},x_{i}^{l,k},y_{i}^{l,k},z_{i}^{l,k}\right) \end{array}\right)\right)}^{2}$$

The Iterative Closest Point approach converges to the optimal solution in an iterative fashion.

#### 2.2.3. Line with Plane Intersection

Each LiDAR beam intersects with the reflective plane. The following plane equation is considered:

(5) ax+by+cz+d=0

where

(6) $$∥\left[\begin{array}{ccc} a & b & c \end{array}\right]∥=1$$

$V^{pl}=\left(a,b,c\right)$ is the unit vector orthogonal to the plane and *d* is the distance from the origin to the plane. It satisfies the following condition with the intersection point in 3D space:

(7) $$\left[\begin{array}{cccc} a & b & c & d \end{array}\right]\left[\begin{array}{c} x\\ y\\ z\\ 1 \end{array}\right]=0$$

The main assumption is that all LiDAR beams starts at origin (0,0,0). Assuming the LiDAR beam has unique representation as beam origin $b^{o}=\left(0,0,0\right)$ and beam direction $b^{d}=\left(b_{x}^{d},b_{y}^{d},b_{z}^{d}\right)$, $∥b^{d}∥=1$ the beam with reflective plane intersection equation is given (8).

(8) $$P^{int}=-\left[\begin{array}{c} b_{x}^{d}\\ b_{y}^{d}\\ b_{z}^{d} \end{array}\right]\frac{d}{V^{pl}\cdot b^{d}}$$

where (·) is the dot product.

#### 2.2.4. Reflection Observation Equation

**Figure 6:**
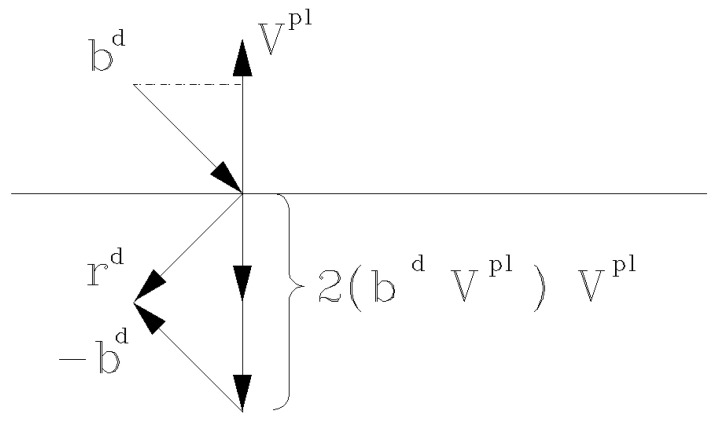
The direction $r^{d}$ of the LiDAR beam $b^{d}$ after reflecting with the plane with normal vector $V^{pl}$.

The direction $r^{d}$ of the LiDAR beam $b^{d}$ after reflecting with the plane with the normal vector $V^{pl}$ is given by Equation (9) and the supportive plot is given in Figure 6.

(9) $$r^{d}=2\left(b^{d}\cdot V^{pl}\right)V^{pl}-b^{d}$$

With the measurement point $P^{l}$($x^{l}$,$y^{l}$,$z^{l}$) expressed in the local coordinate system of the measurement instrument, $l^{p}=∥P^{l}∥$ and distance from origin to intersection $l^{int}=∥P^{int}∥$, the final reflected measurement point $P^{r}$ is given in Equation (10).

(10) $$P^{r}=-\left(P^{int}+r^{d}\left(l^{p}-l^{int}\right)\right)$$

Finally, the measurement point expressed in the global coordinate system is given as (11).

(11) $$P^{g}=\left[R,T\right]P^{r}=\Psi^{r}\left(R,T,a,b,c,d,P^{l}\right)$$

#### 2.2.5. Ground-Truth Data Observation Equation

For ground-truth data as a point cloud composed of points $P^{gt}(x^{gt}$,$y^{gt}$,$z^{gt})$ expressed in global reference systems, the ground-truth data observation equation is given by Equation (12).

(12) $$\begin{array}{c} \underset{residuals}{\underbrace{\left[\begin{array}{c} x^{\delta }\\ y^{\delta }\\ z^{\delta } \end{array}\right]}}=\underset{target\;values}{\underbrace{\left[\begin{array}{c} 0\\ 0\\ 0 \end{array}\right]}}-\underset{model\;function}{\underbrace{\left(\begin{array}{c} \Psi_{\underset{\beta^{j,m}}{\underbrace{\left[R^{j},T^{j},a^{m},b^{m},c^{m},d^{m}\right]}}}^{r}\left(R^{j},T^{j},a^{m},b^{m},c^{m},d^{m},P^{l,j}\right)-\left[\begin{array}{c} x^{gt,k}\\ y^{gt,k}\\ z^{gt,k} \end{array}\right] \end{array}\right)}} \end{array}$$

The optimization process will not modify $P^{gt,k}$. The expected result will converge to obtain minimal distances (target values) between measurement points $P^{l}$ transformed into a global reference system via $\Psi^{r}$ and the corresponding ground-truth $P^{gt}$.

#### 2.2.6. Calibration Algorithm

The angle information from a precise rotating table is used as the additional constraints. The rotation axis of the rotating table and the main optical axis of the calibrated system need to be as close together as possible. Ideally, it should be identical; however, a small discrepancy is acceptable. This geometrical discrepancy is represented by a homogenous transformation $\left[R_{l}^{t},T_{l}^{t}\right]\in SE\left(3\right)$. That homogenous transformation transforms the Livox LiDAR’s local coordinate system to the local coordinate system of the rotating table’s plate. Ideally, it should be the identity, but due to a lack of coaxiality of rotation and optical axis, it represents some unknown, small displacement. Homogenous transformation $\left[R_{l}^{t},T_{l}^{t}\right]$ is treated as an unknown and stationary parameter during calibration. In other words, it is the same for all measurements—Livox LiDAR is rigidly assembled to the rotating table’s plate during calibration. The second homogenous transformation is $\left[R_{t}^{p},T_{t}^{p}\right]\in SE\left(3\right)$, which represents a rotation angle of the rotating table. It is a rotation around the ‘X’ axis with a given, known angle. This homogenous transformation $\left[R_{t}^{p},T_{t}^{p}\right]$ is treated as a known parameter during the calibration process. In our experiment, it is an angle that was set on the rotating table. The third transformation is $\left[R_{p}^{g},T_{p}^{g}\right]\in SE\left(3\right)$. It transforms a stationary measurement station’s local coordinate system to the global coordinate system. It is treated as an unknown parameter during the calibration process. The reflected point ($P^{r}$) in Equation (11) is transformed into the global coordinate system ($P^{g}$) with a chain of transformations:

(13) $$P^{g}=\left[R_{p}^{g},T_{p}^{g}\right]\left[R_{t}^{p},T_{t}^{p}\right]\left[R_{l}^{t},T_{l}^{t}\right]P^{r}$$

Finally, Equation (11), taking into account the chain of SE(3) transforms, has the form:

(14) $$P^{g}=\left[R_{p}^{g},T_{p}^{g}\right]\left[R_{t}^{p},T_{t}^{p}\right]\left[R_{l}^{t},T_{l}^{t}\right]P^{r}=\Psi^{g}\left(R_{p}^{g},T_{p}^{g},R_{t}^{p},T_{t}^{p},R_{l}^{t},T_{l}^{t},a,b,c,d,P^{l}\right)$$

Each of those homogenous transformations belongs to a special Euclidean group SE(3). SE(3) is a Lie group. That means it is possible to represent the given transformation in SE(3) as se(3) algebra. That is a common technique used in the optimization of non-linear problems that contains parameters that belong to SO(3) or SE(3) groups. Further, necessary information can be found in Sola et al. [37]. Properties of Lie algebra se(3) and Lie group SE(3) that are utilized are listed below:

- the point in tangent space to the manifold is a minimal representation (six degrees of freedom).
- the point in tangent space does not have any constraints and can be always exponentially mapped to a valid SE(3).
- every valid SE(3) member can be mapped back to an exact point in tangent space (logarithmic mapping). It is computed using a closed-form solution (Euler–Rodrigues formula).
- every optimized SE(3) transformation contributes six parameters to the optimization problem.

Optimization of all SE(3) parameters is done in the tangent domain. To manipulate an SE(3) and se(3) Sophus C++ library was used [38]. For modelling and optimization of the calibration problem, Ceres solver was used [33]. A mirror is represented in the optimization problem as a plane given with Equation (5). In the calibration process, multiple nearest neighbourhood searches are performed. For this problem the well-known [39] Kd-tree algorithm was utilized. It allows effectively to decompose a point cloud and find the nearest point to a query point.

The calibration algorithm for single measurement station without ground-truth data (the simplest scenario):

- with current calibration point clouds and Kd-trees are built in the global coordinate system.
- using Kd-tree, it searches for pairs of the nearest neighbourhood points that were reflected by a different mirror.
- every found nearest neighborhood point pair $\left(P_{k}^{g},P_{j}^{g}\right)$ creates an observation equation $r_{jk}\in R^{3}$. The point $P_{k}^{g}$ was observed with the measurement $P_{k}^{l}$ (taken in the instrument’s local coordinate system), reflected by the mirror φ and the laser scanner was at rotation ϕ. Mirror φ is represented by its plane parameters via: $a_{\varphi },b_{\varphi },c_{\varphi },d_{\varphi }$. Current rotation of the rotating table is represented with homogenous transformation $\left[R_{l\phi }^{t},T_{l\phi }^{t}\right]\in SE\left(3\right)$. Finally, the residual for the point pair $\left(P_{k}^{g},P_{j}^{g}\right)$ is given by Equation (15).
  (15) $$r_{jk}=P_{j}^{g}-\Psi^{g}\left(R_{l\phi }^{t},T_{l\phi }^{t},R_{l}^{t},T_{l}^{t},a_{\varphi },b_{\varphi },c_{\varphi },d_{\varphi },P_{k}^{l}\right)$$
- Every found pair contributes a new residual. The number of those equations creates an optimization problem. The equation is (15) and is differentiated automatically against all optimized parameters, which are: $R_{l}^{t},T_{l}^{t},a_{\varphi },b_{\varphi },c_{\varphi },d_{\varphi }$
- The optimization problem is solved using the Levenberg–Marquardt algorithm until convergence using the Ceres solver.
- New, the found parameters are applied correctly according to its parametrization and the whole cycle is repeated.

The calibration algorithm for multiple measurement stations uses the stations’ poses $\left[R_{p}^{g},T_{p}^{g}\right]$. In this scenario, if the nearest neighbourhood consists of points from the same measurement station, it will contribute an observation where parameters corresponding to its pose are treated as constant ($\left[R_{p}^{g},T_{p}^{g}\right]$). Otherwise, if the nearest neighbourhood exists between points that were captured from different measurement stations, it will contribute also to these poses an optimization. Finally, if there is ground-truth data the observation Equation (12) can be added. This calibration scenario results in calibration parameters and poses updates; thus, it can be used for 3D map reconstruction.

#### 2.2.7. Calibration Accuracy Evaluation

**Figure 7:**
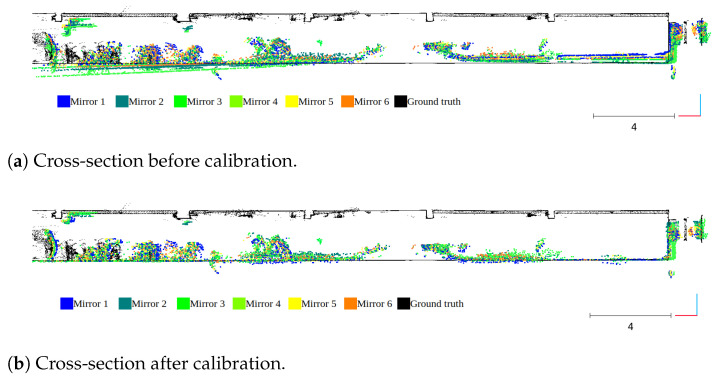
Qualitative result of the calibration impact on improved 3D data accuracy.

**Figure 8:**
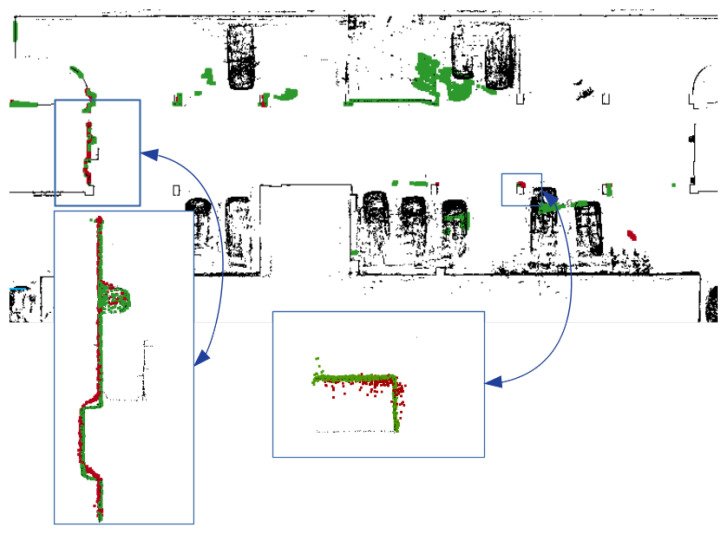
Degradation of LiDAR’s perception with planar reflector. Black—Ground-truth; Green—Livox Mid-40 LiDAR data; Red—Livox Mid-40 LiDAR data with planar reflector. The degradation of sharp corners is evident. The planar target is detected with precision (1σ) 1.8 cm (without reflector) and 2.2 cm (with reflector).

**Figure 9:**
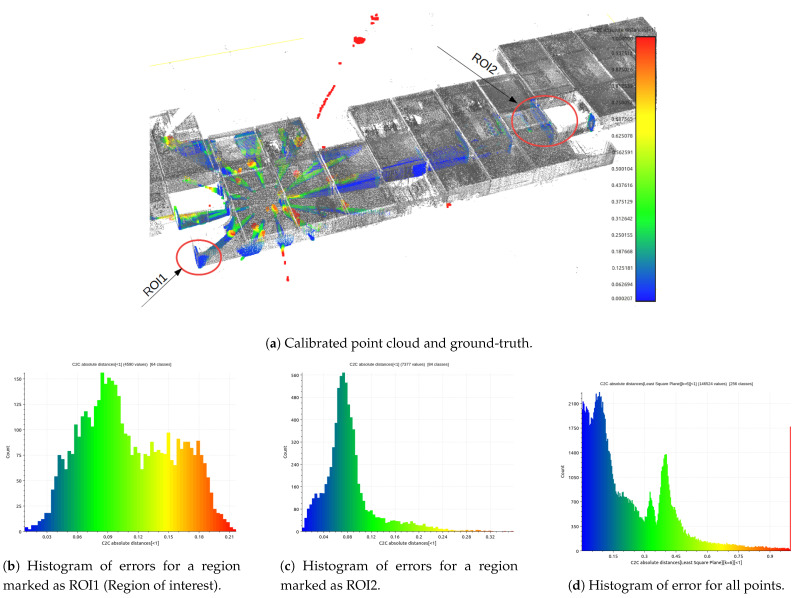
Result of calibration algorithm for single measurement station without ground-truth data (simplest scenario).

**Figure 10:**
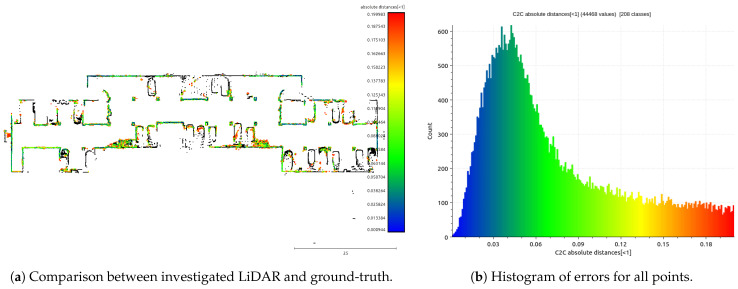
Result of the calibration algorithm for multiple measurement stations with histogram of errors.

**Figure 11:**
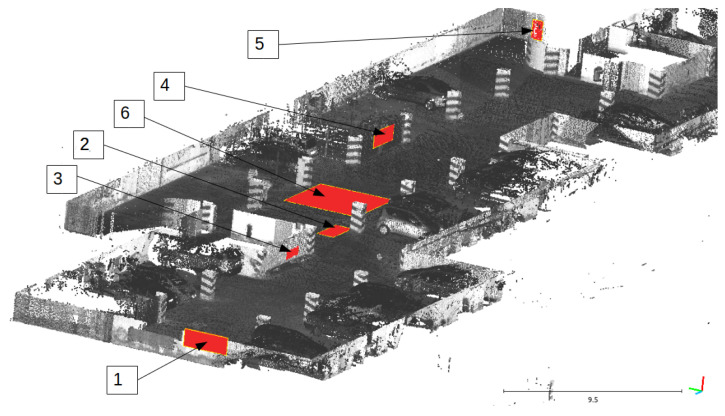
Location of planar targets that are evaluated in Table 2.

The main assumption of the calibration process is the fact that ground-truth data are used only for validation purposes. The evaluation is based on quantitative and qualitative measures of the discrepancy between ground-truth and LiDAR data (Livox Mid-40 with calibrated planar reflectors). The qualitative result is shown in Figure 7 as multiple views of the same features in the scene. The expected outcome of the validation of the calibrated sensor is the consistent view of the cross-section. Moreover, Figure 8 shows the degradation of the identification of the corner shapes. Thus, the negative impact of planar reflectors on LiDAR’s performance is evaluated as the discrepancy between measurements obtained with Livox Mid-40 and the same LiDAR with planar reflectors. Quantitative evaluation is based on measuring error distribution. The error is expressed as the distance of the measured point to its projection onto approximated planar features in ground-truth data. Error distribution is shown in the form of histograms (Figure 9 and Figure 10). Moreover, the quantitative evaluations were performed for planar target detection (Figure 11, Table 2). The goal was to verify the accuracy (measured as mean distance to the target plane) and precision (measured as the standard deviation of distance to the target plane).

## 3. Results

To validate the proposed calibration method the system was tested in a known indoor environment. The environment was mapped with the geodetic measurement instrument Z + F Imager 5010 with accuracy much better than Livox Mid-40. These data are considered as ground truth. Two calibration scenarios were investigated: (1) Single measurement station without ground-truth data; (2) multiple measurement stations. We decided to not add ground-truth data to the optimization process to simulate operational conditions. Ground-truth data are used for qualitative and quantitative evaluation. The qualitative result of the calibration impact on improved 3D data accuracy is shown in Figure 7.

Figure 9 shows the results of the calibration algorithm for a single measurement station without ground-truth data (simplest scenario). Figure 10 shows the results for multiple measurement stations. We observed that calibration of planar reflectors can be done based on a single measurement station. Better calibration results can be reached for multiple measurement stations, but it is a more complicated procedure requiring more time for data acquisition. The better calibration outcome can be seen comparing histograms Figure 9a and Figure 10b. Calibration found with multiple measurement stations has unimodal distribution of error and the most common error value is smaller. Calibration with multiple measurement stations is more robust and performs significantly better. Calibration with single measurement stations is much easier to perform, although it is susceptible to convergence to a sub-optimal solution. Both distributions of the errors (Figure 9a and Figure 10b) are Gaussian with large positive skewness. Long-tail is present in distributions. Both are caused by outliers. Deviation of the optimized parameters before and after calibration is shown in Table 3. For example, the angular update of the first planar reflector is 0.65${}^{{}^{\circ}}$. This is an important insight into the calibration impact. Unfortunately, planar reflectors generate outlier points and some artifacts affecting point cloud quality. This is shown in Figure 8. The accuracy and the precision were evaluated based on six planar ground-truth targets shown in Figure 11. The results confirm minor degradation of the accuracy and the precision compared with reported parameters by the Livox vendor (Table 1 [29]).

For this reason, in future work, we will focus on filtering algorithms.

## 4. Discussion

The calibration process of planar reflectors reshaping LiDAR’s field of view enables preserving accuracy and precision of the LiDAR to some extend. Based on our best knowledge such an investigation has not yet been discussed in the literature. We demonstrated by the experiment that the calibration process is required for affordable solutions where the mechanical design can differ from the inaccurate assembly. We use a state-of-the-art LiDAR Livox Mid-40 sensor as an object of investigation of the reshaping field of view with planar reflectors. Ground-truth data were collected with a precise Z + F IMAGER 5010 terrestrial laser scanning system. The calibration process incorporates the state-of-the-art reflection observation equation integrated with Iterative Closest Point optimization. We observed that adding planar reflectors slightly degrades the precision of the measurements; in particular, detected corners are slightly curved. This observation yields the conclusion that such a sensor will be rather useful for localization purposes than environmental mapping. Moreover, future autonomous mobile robots will require such calibration if their LiDAR FOV will be reshaped by planar reflectors.

The impact of planar reflectors on another popular LIDAR (Velodyne VLP-16) was investigated in detail in the paper [19]. In their work, the authors investigated power loss and LiDAR’s scanning pattern after FOV reshaping. They attempted to reshape the original omnidirectional FOV to a narrow FOV maximizing the angular resolution of the system. They performed simulations and examined the built prototype. They concluded that FOV reshaping using a planar reflector is feasible and yields a reduction in the range of 3.9% (when the incident angle to the target is preserved). In our work, we built a similar setup using alike technology of prototyping, but we investigated the unaddressed issue of planar reflector calibration. We also introduced a slightly different LiDAR to that used in the discussed paper. The difference between Velodyne and Livox Mid-40 LiDARs can be found in the paper [2].

Our work contributes to omnidirectional perception based on 3D LiDAR sensor similarly to [40]. The authors developed a 3D laser scanner based on a SICK LMS 200 LiDAR, which consists of the LMS 200 facing upwards into a rotating mirror driven by a stepper motor. It is worth mentioning the work [41] ona 3D imaging LiDAR based on the high-speed 2D laser scanner and the work [42] on the automatic calibration of spinning actuated LiDAR internal parameters. These approaches relate also to omnidirectional perception derived from spinning LiDARs. Such an approach is not affordable; moreover, introducing other moving parts into the design decreases the robustness of the entire system. A recent survey [43] of low-cost 3D laser scanning technology discusses other approaches; unfortunately, the calibration of the planar reflectors reshaping LiDAR’s field of view is not elaborated. For this reason, we are focused on this aspect in this paper.

## 5. Conclusions

This paper describes the calibration process of planar reflectors reshaping LiDAR’s field of view. The calibration method is based on the reflection equation used in the Iterative Closest Point optimization. The final metric measurement is more accurate compared with parameters retrieved from the mechanical design. The experiment is performed based on Livox Mid-40 LiDAR augmented with six planar reflectors. The ground-truth data were collected using Z + F IMAGER 5010 3D Terrestrial Laser Scanner. We show two scenarios: (1) Single measurement station without ground-truth data, (2) multiple measurement stations. It is documented by an experiment that the scenario of multiple measurement stations gives better qualitative and quantitative results compared with a single measurement station. However, the procedure requires greater effort in data acquisition. Moreover, this multi-view data registration scheme enables optimizing poses and parameters of planar reflectors simultaneously. It can be used for 3D map reconstruction. The calibration method is independent of mechanical design and does not require any fiducial markers on the mirrors. This work fulfills the gap between rotating and Solid-State LiDARs since the field of view can be reshaped by planar reflectors, and the proposed method can preserve the metric accuracy. There are many disadvantages of additional planar reflectors such as reducing the number of useful data and the negative impact on the sensor’s range. We observed a decreased range of the Livox Mid-40 LiDAR, even down to 80 per cent. The important finding is the degradation of LiDAR’s perception with a planar reflector. The degradation of sharp corners is evident. In contrast, an interesting fact is that the precision of the detection of the planar shape is rather similar; thus, an example planar target was detected with precision (1σ) 1.8 cm (without reflector) and 2.2 cm (with reflector). These quantitative measures confirm the nominal range precision reported by the Livox manufacturer. This work can be applied for multi LiDAR sensor calibration and other practical scenarios, e.g., 3D digitization of the urban environment, cultural heritage and archaeology, underground environment, environmental monitoring, forestry and agriculture assuming the usage of an affordable LiDAR with the reshaped field of view with planar reflectors. In future work, we will focus on localization aspects using the proposed 3D LiDAR prototype since it is an affordable, wide field of view and a long-range solution does not exist in the market and is not discussed in the literature. We prepared an open-source project and provided all the necessary data, including software, CAD design and sample captures for reproducing the experiments.

## Figures and Tables

**Table 1:** Livox Mid-40 [29] and Z + F IMAGER 5010 [30] parameters. (*) Measured in an environment of 25 ${}^{{}^{\circ}}$C with a target (80% reflectivity) 20 m away. The result may vary under different test conditions.

Parameter	Livox Mid-40	Z + F IMAGER 5010
Laser Wavelength	905 nm	1500 nm
Laser Safety	Class 1 (IEC60825-1)	Class 1
Detection Range	(@ 100 klx) 90 m @ 10% reflectivity	187.3 m
	130 m @ 20% reflectivity	
	260 m @ 80% reflectivity	
FOV	38.4${}^{{}^{\circ}}$ Circular	320${}^{{}^{\circ}}$ Vertical, 360${}^{{}^{\circ}}$ Horizontal
Range Precision	(1σ @ 20 m) 2 cm (*)	1 mm

**Table 2:** The evaluation of accuracy and precision of planar targets detection using Livox Mid-40 with planar reflector. Locations of planar targets are shown in Figure 11.

Plane Number	Accuracy [cm] (Mean Distance to the Plane)	Precision [cm] (Standard Deviation of Distance)	Distance to Sensor [m]
1	2.95	1.16	18.3
2	0.32	2.01	20.2
3	0.22	1.26	15.5
4	2.07	2.73	35.7
5	3.26	3.05	60.3
6	2.01	1.20	24.5

**Table 3:** The optimized parameters (planar reflectors’ coefficients); u: Unitless quantity; m: Meter; [a,b,c]: Unit vector of planar reflector; *d*: Distance of planar reflector to the origin.

Parameter	Initial	Calibrated
$\left[a_{1},b_{1},c_{1},d_{1}\right]$	[0.793 u, −0.304 u, −0.527 u, −0.075 m]	[0.799 u, −0.311 u, −0.519 u, −0.044 m]
$\left[a_{2},b_{2},c_{2},d_{2}\right]$	[−0.793 u, 0.609 u, −0.000 u, 0.075 m]	[−0.787u,0.614u, −0.012u,0.049m]
$\left[a_{3},b_{3},c_{3},d_{3}\right]$	[0.793 u, −0.304 u, 0.527 u, −0.075 m]	[0.791 u, −0.301 u, 0.531 u, −0.049 m]
$\left[a_{4},b_{4},c_{4},d_{4}\right]$	[0.793 u, 0.304 u, 0.527 u, −0.075 m]	[0.789 u, 0.311 u, 0.528 u, −0.049 m]
$\left[a_{5},b_{5},c_{5},d_{5}\right]$	[−0.793 u, −0.609 u, −0.000 u, 0.075 m]	[−0.798 u, −0.605 u, 0.008 u, 0.045 m]
$\left[a_{6},b_{6},c_{6},d_{6}\right]$	[0.793 u, 0.304 u, −0.527u, −0.075 m]	[0.793 u, 0.291 u, −0.534 u, −0.047 m]

## Data Availability

Publicly available datasets were analyzed in this study. This data can be found here: https://github.com/michalpelka/catoptric_livox [32].

## Abbreviations

The following abbreviations are used in this manuscript:

CAD	Computer Aided Design
FDM	Fused Deposition Modeling
GPS	Global Positioning System
FOV	Global Positioning System
ICP	Iterative Closest Point
LiDAR	Light Detection and Ranging
MEMS	Micro Electro Mechanical Systems
PLA	PolyLactic Acid
PMMA	PolyMethyl MethAcrylate
SLAM	Simultaneous Localization and Mapping
ROI	Region of iterest
